# Supplementary material for: Antibacterial and Analgesic Properties of Beta-Caryophyllene in a Murine Urinary Tract Infection Model
Source: Molecules. 2023 May 17;28(10):4144. doi: 10.3390/molecules28104144 (PMC10223983; doi:10.3390/molecules28104144)
Supplement: Supplementary file 1 [file molecules-28-04144-s001.zip › molecules-2327472-supplementary.docx]

**Supplementary Materials:** The following supporting information can be downloaded at: www.mdpi.com/xxx/s1, Table S1; Figure S1; Figure S2; Figure S3; Figure S4.

**Table 1.** Murine behavioral scoring parameters.

| **Parameter** | **Description** |
| --- | --- |
| Eye opening | 0 complete opening, 5 half opening, 10 full closed, 2 and 7 are intermediate positions |
| Posture | No change is 0, full rounded back OR limp is 10. Intermediate specific postures gain other scores |
| Motor activity | Activity (exploring, moving, grooming) within 20 seconds. E.g. no movement for 10 seconds (=50%) = 5 points; no movement for 2 seconds (=10%) = 1 point |

^1^ Behavioral scoring criteria adapted from Boucher et al.[27]. Each parameter gains a score between one and ten, to a maximum score of 30 overall.

**Supplemental Figure 1.** Representative histology images are showing features of inflammation. Panels A/B shows a representative score of 0 taken from a Sham (uninfected) animal, with blood vessel confined immune cells (A; 400x). Cells were identified to be primarily neutrophils (B; 1000x). Panels C/D show a bladder sample (score=0.75) from a UPEC infected mouse, with focal collections of immune cells further away from the blood vessels (C). Cells were primarily neutrophils and lymphocytes (D). Tissue damage scores, as assessed by hematoxylin and eosin staining of bladder tissue of mice six and 24 hours post induction of UTI (E). Data presented as mean ± SD, * *p*<0.05

**Supplemental Figure 2.** Changes in cytokine levels in the bladder tissue, measured by multiplex cytokine assay, as assessed in mice six and 24 hours post induction of UTI. Cytokines measured included IL-6 (A; undetectable at 24 hours), CXCL2 (B; undetectable at 24 hours), P-selectin (C), I-CAM-1 (D), IL-10 (E), and LIX (F). Data presented as mean ± SD, * *p*<0.05

**Supplemental Figure 3.** Changes in the evoked pain threshold, measured via von Frey aesthesiometry, as assessed in mice six (A), 24 (B) and 72 hours (C) post induction of UTI. Data presented as mean ± SD, * *p*<0.05
